# Structure of bovine cytochrome *c* oxidase crystallized at a neutral pH using a fluorinated detergent

**DOI:** 10.1107/S2053230X17008834

**Published:** 2017-06-20

**Authors:** Fangjia Luo, Kyoko Shinzawa-Itoh, Kaede Hagimoto, Atsuhiro Shimada, Satoru Shimada, Eiki Yamashita, Shinya Yoshikawa, Tomitake Tsukihara

**Affiliations:** a Picobiology Institute, Graduate School of Life Science, University of Hyogo, 3-2-1 Koto, Kamigori-cho, Ako-gun, Hyogo 678-1297, Japan; b Graduate School of Applied Biological Sciences and Faculty of Applied Biological Sciences, Gifu University, 1-1 Yanagido, Gifu 501-1193, Japan; c Institute for Protein Research, Osaka University, 3-2 Yamadaoka, Suita, Osaka 565-0871, Japan; d Japan Science and Technology Agency, CREST, 4-1-8 Honcho, Kawaguchi, Saitama 332-0012, Japan

**Keywords:** cytochrome *c* oxidase, membrane-protein complex, X-ray structure, neutral pH

## Abstract

Cytochrome *c* oxidase (CcO) couples proton pumping to O_2_ reduction. Its enzymatic activity depends sensitively on pH over a wide range. However, owing to difficulty in crystallizing this protein, X-ray structure analyses of bovine CcO aimed at understanding its reaction mechanism have been conducted using crystals prepared at pH 5.7, which is significantly lower than that in the cell. Here, oxidized CcO at pH 7.3 was crystallized using a fluorinated octyl-maltoside derivative, and the structure was determined at 1.77 Å resolution. No structural differences between crystals obtained at the neutral pH and the acidic pH were detected within the molecules. On the other hand, some differences in intermolecular interactions were detected between the two types of crystal. The influence of pH on the molecular surface is likely to contribute to the pH dependency of the aerobic oxidation of ferrocytochrome *c*.

## Abstract 2

Although the enzymatic activity of cytochrome *c* oxidase (CcO) depends sensitively on pH over a wide range, X-ray structure analyses of bovine CcO have been conducted using crystals prepared at pH 5.7 owing to difficulty in crystallizing this protein. Here, oxidized CcO at pH 7.3 was successfully crystallized using a fluorinated octyl-maltoside derivative, and the structure was determined at 1.77 Å resolution.

## 1. Introduction   

Cytochrome *c* oxidase (CcO), the terminal oxidase in cellular respiration, couples O_2_ reduction to proton pumping. Mammalian CcO is located in the inner mitochondrial membrane and accepts electrons from cytochrome *c* on the P-side. The protons used in H_2_O formation are supplied to the enzyme through the D- and K-pathways from the N-side, whereas pumped protons are transferred through the enzyme from the N-side to the P-side in order to generate a proton-concentration gradient (ΔpH) across the inner mitochondrial membrane (Yoshikawa & Shimada, 2015 ▸). Bovine CcO is a large transmembrane protein that exists as a 420 kDa dimer in its crystalline state. Following the structure determination of four redox-active metal sites [haem *a* (Fe*_a_*), haem *a*_3_ (Fe_*a*3_), Cu_B_ and Cu_A_; Tsukihara *et al.*, 1995 ▸], as well as the overall protein structure of the oxidized state (Tsukihara *et al.*, 1996 ▸), structural changes around Asp51 of subunit I [Asp51(I)] that occur upon reduction of the CcO crystal were detected at 2.3 Å resolution. In addition, these analyses identified a connection between the residue on the P-side surface and the N-side surface *via* a proton-conducting pathway (Yoshikawa *et al.*, 1998 ▸), later named the H-pathway. Based on these structural findings, it was proposed that protons are pumped through the H-pathway, including Asp51(I).

Additional high-resolution X-ray structure analyses of the oxidized and reduced states revealed various redox-coupled structural differences in helix X (Muramoto *et al.*, 2010 ▸), haem *a*, haem *a*_3_, Arg438(I), Glu198(II) and water molecules around the Mg-ion site in subunit I (Yano *et al.*, 2016 ▸).

The H-pathway, through which protons are actively transferred, is composed of a water channel and a hydrogen-bond network that operate in tandem. The water channel in the H-pathway includes spaces, designated as water cavities, in which at least one mobile water is stored to promote overall efficiency in proton transfer and water-molecule exchange through the water channel (Yano *et al.*, 2016 ▸). The hydrogen-bond network is connected to a proton-storage site *via* a short hydrogen-bond network (see Fig. 3 of Yano *et al.*, 2016 ▸). The storage site, which consists of an Mg ion, water molecules and several proton-accepting residues, including Glu198(II) and Arg438(I), designated as the Mg-containing water cluster, saves four protons to be pumped (Yano *et al.*, 2016 ▸). The Mg-containing water cluster collects all four protons in the fully reduced state through the open water channel. A structural change in helix X affects the sizes of the water cavities, resulting in closure of the water channel (Muramoto *et al.*, 2010 ▸), which is triggered by the translational shift of the haem *a*_3_ plane upon O_2_ binding to CcO after the four protons have been collected (Yano *et al.*, 2016 ▸).

A proton-pumping mechanism involving the D-pathway, instead of the H-pathway, has been proposed based on mutational analyses of bacterial CcOs, the structures of which are very similar to those of mammalian CcOs (Yoshikawa & Shimada, 2015 ▸). To date, however, no redox-coupled X-ray structural change of bovine CcO supporting the D-pathway proposal has been observed, and the structure of the D-pathway is insensitive to the oxidation states of any of the redox-active metal sites.

Owing to the pH sensitivity of the crystallization conditions of bovine heart CcO, all previously solved structures of CcO were determined using crystals formed at pH 5.7. However, it is necessary to determine the X-ray structure at neutral pH in order to obtain structural insights under physiological conditions. Because crystallization conditions for bovine CcO under the proton gradient have not been established, we sought to determine the structure of CcO at neutral pH using crystals prepared using a mild detergent, in order to obtain structural insights under physiological conditions.

## 2. Materials and methods   

### 2.1. Preparation of CcO crystals in the fully oxidized state   

CcO in the fully oxidized state was purified from bovine heart mitochondria (Tsukihara *et al.*, 1995 ▸) and was dissolved in 40 m*M* sodium phosphate buffer pH 6.8 containing 0.20%(*w*/*v*) *n*-decyl-β-d-maltoside (decyl maltoside; Dojin). CcO was diluted tenfold in 25 m*M* Tris–HCl buffer pH 7.3 containing 33 m*M* sodium acetate, 0.20%(*w*/*v*) decyl maltoside and 0.70%(*w*/*v*) fluorinated octyl-maltoside [also known as 3,3,4,4,5,5,6,6,7,7,8,8,8-tridecafluoro-*n*-octyl-β-d-maltopyranoside (FOM)] (Anatrace). FOM-treated CcO was concentrated to 100 mg ml^−1^ using a membrane filter (Amicon Ultra centrifugal filters, 100 kDa, Millipore). Crystallization of oxidized CcO dissolved in decyl maltoside and FOM was performed by a batch method at 277 K. CcO was mixed with a precipitant, polyethylene glycol (PEG) 1500, at a concentration of ∼3%. CcO crystals were obtained as rectangular plates within 1 d. The crystals were gradually soaked in a solution containing ethylene glycol (EG) as a cryoprotectant, reaching final concentrations of 40% EG and 10% PEG 1500. After equilibration in the cryoprotectant solution, the crystals were flash-cooled in a nitrogen stream at 100 K.

### 2.2. X-ray diffraction experiments   

All X-ray experiments were carried out on beamline BL44XU at SPring-8 equipped with an MX225-HE CCD detector. Crystals with dimensions of ∼700 × 700 × 200 µm were used for the diffraction experiments. The thin edge of a crystal was aligned parallel to the X-ray beam at a rotation angle of 0.0° for all diffraction experiments. The wavelength was 0.9 Å, and the dose at the sample position was 4.0 × 10^11^ photons s^−1^. Each crystal was exposed to X-rays in a helium-gas stream at 50 K and translated by 10 µm after each exposure to reduce radiation damage. Other experimental conditions used for collecting low-resolution data were as follows: an X-ray beam cross-section of 20 µm (vertical) × 20 µm (horizontal) at the crystal, a camera distance of 431 mm, an exposure period of 1.0 s and an oscillation angle of 1.0°. The conditions for collecting high-resolution data were as follows: an X-ray beam cross-section of 50 µm (vertical) × 30 µm (horizontal) at the crystal, a camera distance of 230 mm, an exposure period of 3.0 s and an oscillation angle of 0.5°. A total of four crystals were used to acquire full data sets at a resolution of 1.77 Å. The radiation dose for each diffraction experiment was estimated using *RADDOSE* (Murray *et al.*, 2004 ▸). Data processing and scaling were carried out using *HKL*-2000 (Otwinowski & Minor, 1997 ▸). A total of 586 images were successfully processed and scaled. Structure-factor amplitudes (|*F*|) were calculated using the *CCP*4 program *TRUNCATE* (French & Wilson, 1978 ▸; Weiss, 2001 ▸). The statistics for the intensity data are provided in Table 1 ▸.

### 2.3. Structure determination and refinement   

The same procedures as applied in previous structural analyses of CcO crystals obtained at pH 5.7 (Yano *et al.*, 2016 ▸) were followed for structure determination. The structure determination was initiated by the molecular-replacement (MR) method (Rossmann & Blow, 1962 ▸) using the *CCP*4 program *MOLREP* (Vaguine *et al.*, 1999 ▸). Initial phases of structure factors up to a resolution of 4.0 Å were calculated by the MR method using a model built from a fully oxidized structure previously determined at 1.8 Å resolution (PDB entry 1v54; Tsukihara *et al.*, 2003 ▸) after removing nonprotein molecules including waters, lipids and detergents. The phases were extended to a resolution of 1.77 Å by density modification (Wang, 1985 ▸) coupled with noncrystallographic symmetry (NCS) averaging (Bricogne, 1974 ▸, 1976 ▸) using the *CCP*4 program *DM* (Cowtan, 1994 ▸). The resultant phases (α_MR/DM_) were used to calculate the electron-density map (MR/DM map) with Fourier coefficients |*F*_o_|exp(*i*α_MR/DM_)*,* where |*F*_o_| is the amplitude of the observed structure factor. A difference electron-density map with coefficients (|*F*_pH5.7_| − |*F*_pH7.3_|)exp(*i*α_pH5.7_) was calculated to compare the structures obtained at pH 7.3 and pH 5.7, where |*F*_pH5.7_| and α_pH5.7_ are the observed structure-factor amplitudes and phases of the crystal at pH 5.7 previously determined at 1.5 Å resolution (Yano *et al.*, 2016 ▸), respectively.

Bulk-solvent correction and anisotropic scaling of the observed and calculated structure-factor amplitudes, as well as TLS parameters, were incorporated into the refinement calculation using *REFMAC* (Murshudov *et al.*, 2011 ▸). Anisotropic temperature factors for the Fe, Cu and Zn atoms were imposed in the calculation of structure factors. Each monomer of pairs related by NCS was assigned to a single TLS group during the refinement. The crystal structure was refined under NCS restraints between two monomers. Water molecules, ethylene glycols, lipids and detergents were located in the MR/DM and (|*F*_o_| − |*F*_c_|) maps after refinement. Occupancies of water O atoms in the Mg-containing cluster were determined by adjusting the *B* factors according to previous structural analyses (Yano *et al.*, 2016 ▸).

## 3. Results and discussion   

### 3.1. Crystallization at pH 7.3 and intensity data acquisition   

We were able to reproducibly grow large rectangular crystals using detergent conditions that included 0.20%(*w*/*v*) decyl maltoside and 0.70%(*w*/*v*) FOM. The mild detergent FOM enabled the successful crystallization of CcO at pH 7.3, as in the case of the cytochrome *c*–CcO complex at pH 8.0. The crystal data, as well as the intensity data statistics, are given in Table 1 ▸. The statistics *R*_merge_, *R*_p.i.m._ and *I*/σ(*I*), as well as the completeness and averaged redundancy, indicated that the intensity data were of high quality at a resolution of 1.77 Å. Average radiation doses ranged between 0.43 and 0.45 MGy for all rotation angles.

### 3.2. Structure determination and refinement   

Structure-refinement statistics are listed in Table 2 ▸. The refinement converged well to an *R*_work_ of 0.164, an *R*_free_ of 0.191, a root-mean-square deviation (r.m.s.d.) for bond lengths of 0.011 Å and an r.m.s.d. for bond angles of 1.20°. Of 3614 protein residues, the structures of 64 residues, residues 1–2 of subunit III, 1–3 of subunit IV, 1–4 of subunit Va, 95–98 of subunit Vb, 85 of subunit VIa, 1–6 of subunit VIb, 59 of subunit VIIa, 1–5 and 55–56 of subunit VIIb, 1 of subunit VIIc and 44–46 of subunit VIII for both monomers *A* and *B*, were not built in the electron-density maps owing to poor electron-density distribution, where monomers *A* and *B* consist of subunits *A*–*M* and *N*–*Z* in the PDB definition, respectively. Other flexible regions with high *B* factors are as follows: residues 4–8 of subunit IV in monomer *B*, 1–11 of subunit VIa in both monomers, 7–9 of subunit VIb in both monomers and 1–2 of subunit VIc in monomer *B*. We found that 125 residues were in multiple conformations. We assigned 34 lipid, 14 detergent, 2885 water and 56 ethylene glycol molecules. The averaged *B* factors of protein atoms were estimated for two crystallo­graphically independent monomers *A* and *B*. The *B* factors for monomers *A* and *B* are 36.7 and 44.4 Å^2^, respectively. Because the structures of both monomers in the asymmetric unit of the crystal were refined under NCS restraints, and monomer *A*, which had the lower *B* factor, had higher quality atomic coordinates than monomer *B*, we chose to describe the structural features of monomer *A*.

### 3.3. Structural similarities to the structure at pH 5.7   

**Figure 1:**
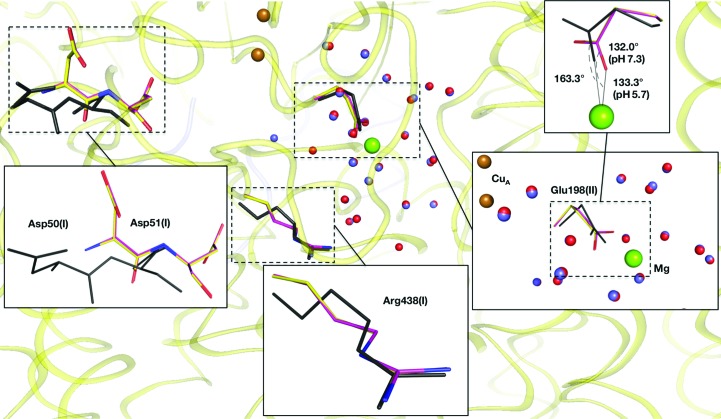
Structures of the Mg-containing water cluster and Asp50-Asp51(I) for the oxidized crystals at pH 7.3 and pH 5.7, and the reduced crystal at pH 5.7, are shown as close-up views (boxes). Amino-acid residues are shown as sticks. Their N atoms are coloured blue, and C atoms of the oxidized crystals at pH 7.3 and pH 5.7 are coloured pink and yellow, respectively. The amino-acid residues of both oxidized crystals superpose well on each other. Residues of the reduced crystal at pH 5.7, drawn as thin black sticks, differ structurally from the residues of the oxidized crystals. The green and brown spheres are Mg and Cu ions, respectively. The red and blue spheres are waters belonging to the oxidized crystals at pH 5.7 and pH 7.3, respectively. Glu198(II) bridging the Cu_A_ and Mg ions changes its coordination angle to the Mg ion upon redox change.

The (|*F*_pH5.7_| − |*F*_pH7.3_|) map calculated at 1.77 Å resolution did not reveal any structural differences around the Mg-containing water cluster between the crystals generated at pH 7.3 and pH 5.7. The structures of Asp51(I), Arg438(I) and Glu198(II), which are sensitive to redox changes of the metal centres at pH 5.7, are almost identical to those of the oxidized crystal at pH 5.7 (Fig. 1 ▸). A total of 24 sites in the Mg-containing water cluster were assigned as waters in the MR/DM and (|*F*_o_| − |*F*_c_|) maps. Two sites were as close as 1.24 Å to each other, as previously observed in the low-pH crystals. The two sites were assigned as multiple sites that did not coexist in one molecule. All of the water sites in the Mg-containing water cluster are in almost the same positions as those in the corresponding states at pH 5.7. Following our previous paper (Yano *et al.*, 2016 ▸), we determined the occupancies of water molecules in the Mg-containing water cluster, and found that the total occupancy of water molecules was 21.15; this number is identical to that of the low-pH crystal within experimental error (Table 3 ▸).

### 3.4. Intermolecular interactions within the crystals differ at pH 7.3 *versus* pH 5.7   

**Figure 2:**
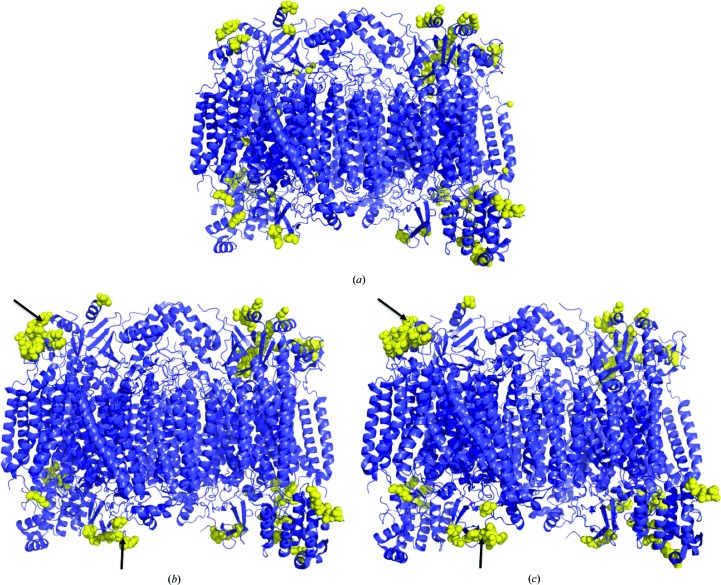
Intermolecular interactions within the crystals. CcO dimers are drawn as ribbon models in blue. Yellow spheres are residues interacting with different molecules within 4.0 Å in each crystal. (*a*) Oxidized state at pH 7.3. (*b*) Oxidized state at pH 5.7. (*c*) Reduced state at pH 5.7. Residues 112, 114, 115 and 118 of subunit IV of monomer *B* (*Q* in the PDB definition), indicated by the top arrow, and residues 93, 94, 95 and 98 of subunit Vb of monomer *B* (*S* in the PDB definition), indicated by the bottom arrow, interact closely with other molecules in the pH 5.7 crystals.

Residues in the CcO dimer that interact with other CcOs in the crystal are shown in Figs. 2 ▸(*a*), 2 ▸(*b*) and 2 ▸(*c*) for the oxidized crystal at pH 7.3, the oxidized crystal at pH 5.7 and the reduced crystal at pH 5.7, respectively. The oxidized and reduced crystals are almost identical to each other in the intermolecular interactions in the crystals. Although the three crystals are isomorphous to each other, some differences are present between the crystals at pH 7.3 and pH 5.7 in the intermolecular interactions in the crystals. Residues 112, 114, 115 and 118 of subunit IV (*Q* in the PDB definition) interact closely with other molecules in the pH 5.7 crystals, whereas in the pH 7.3 crystals these residues make no contacts closer than 4.0 Å. Residues 93, 94, 95 and 98 of subunit Vb (*S* in the PDB definition) made intermolecular contacts within 4.0 Å in the pH 5.7 crystals, whereas these residues were disordered in the pH 7.3 crystals. The difference in the intermolecular inter­action between the pH 7.3 and pH 5.7 crystals indicated differences in the nature of the molecular surfaces of the molecules. The influence of pH on the molecular surface is likely to contribute to the pH dependency of the enzyme activity as determined by the subsequent aerobic oxidation of ferrocytochrome *c*, the rate of which is limited by the interaction between the protein moieties of cytochrome *c* and CcO (Yonetani & Ray, 1995 ▸).

### 3.5. Detergents in the crystals   

Eight molecules of cholate, which was used for extraction of CcO from the mitochondrial inner membrane, were identified in each MR/DM and (|*F*_o_| − |*F*_c_|) map. All cholate sites were shared between both pH states of the crystals. Four models of decyl maltoside in the oxidized and reduced states were built at the same sites as those of the pH 5.7 crystal in the MR/DM and (|*F*_o_| − |*F*_c_|) maps. The hydrocarbon tail of each decyl maltoside is in the hydrophobic region of the CcO molecule, and its terminal rings are exposed outside the CcO molecule. Furthermore, an additional electron density, assignable to either a decyl maltoside or an FOM from the crystallization solution at pH 7.3, was detected on the molecular surface. Several iterations of refinement were conducted assigning the electron density as FOM. Because the refinement provided abnormally high *B* factors for the tail atoms of FOM, these electron densities were assigned as decyl maltosides. No decyl maltoside was replaced by FOM, as in the case of the cytochrome *c*–CcO complex crystallized at pH 8.0 using decyl maltoside and FOM (Shimada *et al.*, 2017 ▸), which was likely owing to the lower hydrophobicity of the fluorinated tail in comparison with that of the corresponding hydrocarbon tail.

### 3.6. The structure of the dioxygen-reduction centre   

**Figure 3:**
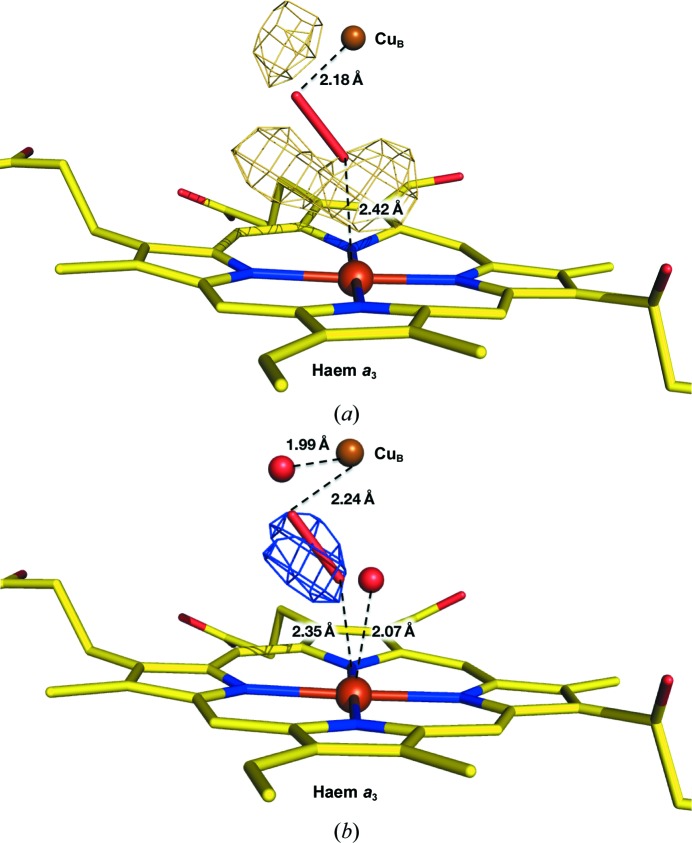
Difference maps calculated with coefficients (|*F*_o_| − |*F*_c_|)exp(*i*α_c_). Haems *a*_3_ are drawn as stick models, with C, N and O atoms coloured yellow, blue and red, respectively. Fe*_a_*_3_ and Cu_B_ are shown as red and brown spheres, respectively. A peroxide ion is shown as a stick with a bond length of 1.50 Å. All difference maps were drawn at the 3.0σ level. (*a*) The difference map for refinement with the peroxide bond length constrained to 1.50 Å. The residual density map indicates two peaks each close to Cu_B_ and Fe_*a*3_. These peaks were assigned as partially occupied O atoms. (*b*) The difference map calculated with the peroxide bond length constrained to 1.50 Å and the occupancies of the peroxide and a pair of O atoms set to 90 and 10%, respectively. The residual density in the dioxygen-reduction centre is significantly lower than in the other cases.

An (|*F*_o_| − |*F*_c_|) map with phases calculated by including a peroxide ligand with a bond length of 1.50 Å had significant residual densities in the dioxygen-reduction centre of the oxidized form (Fig. 3 ▸ *a*). The residual densities suggested the presence of two additional O atoms close to Fe*_a_*_3_ and Cu_B_, respectively, and a translational shift for the peroxide. The residual densities of the (|*F*_o_| − |*F*_c_|) map were significantly reduced when the structure was refined with 90% occupancy for the peroxide and 10% occupancy for the two O atoms (Fig. 3 ▸ *b*). Consequently, in addition to the bridging peroxide, two O atoms, each with 10% occupancy, were identified close to Fe*_a_*_3_ and Cu_B_ at distances of 2.07 and 1.99 Å, respectively. Because these two O atoms did not exist in the structure of the oxidized form determined by XFEL radiation damage-free structure analysis (Hirata *et al.*, 2016 ▸), it is likely that these two O atoms are generated from the peroxide by X-ray irradiation.

## 4. Conclusion   

We have conducted X-ray structure analyses of bovine CcO aimed at understanding its reaction mechanism using crystals prepared at pH 5.7, which is significantly lower than that in the cell. X-ray structures of CcO crystals at pH 5.7 suggest that the proton pumping is regulated by structural changes within the protein molecule during the reaction cycle (Muramoto *et al.*, 2010 ▸; Tsukihara *et al.*, 1996 ▸, 2003 ▸; Yano *et al.*, 2016 ▸; Yoshikawa *et al.*, 1998 ▸). No structural differences between crystals obtained at neutral pH and acidic pH were detected within the molecule. Thus, X-ray structures of various states at pH 5.7 are available for studies to understand the reaction mechanism of CcO.

## Supplementary Material

PDB reference: bovine heart cytochrome *c* oxidase, 5xdq

## Figures and Tables

**Table 1:** X-ray diffraction data for oxidized CcO at neutral pH Values in parentheses are for the highest resolution shell.

Beamline	BL44XU, SPring-8
Oscillation angle (°)	0.5
Resolution (Å)	200–1.77 (1.79–1.77)
Wavelength (Å)	0.9
Space group	*P*2_1_2_1_2_1_
Unit-cell parameters (Å)	*a* = 177.53, *b* = 183.31, *c* = 205.90
No. of images	586
No. of crystals	4
Observed reflections	5739764
Independent reflections	643861 (16039)
Multiplicity	8.9 (8.2)
Completeness (%)	99.9 (100.0)
〈*I*/σ(*I*)〉	31.2 (3.3)
Wilson *B* factor (Å^2^)	30.7
*R*_merge_ † (%)	8.6 (>100)
*R*_p.i.m._ ‡ (%)	3.2 (38.6)

† *R*_merge_ = 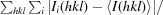 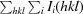, where *I_i_*(*hkl*) is the intensity value of the *i*th measurement of *hkl* and 〈*I*(*hkl*)〉 is the corresponding mean value of *I_i_*(*hkl*) for all *i* measurements. The summation is over reflections with *I*/σ(*I*) larger than −3.0.

‡ *R*_p.i.m._ = 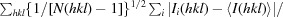 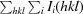, where *N*(*hkl*) is the multiplicity of each *hkl*.

**Table 2:** Statistics of structure refinement of oxidized CcO at neutral pH

Resolution (Å)	200–1.77
*R*_work_ (%)	16.39
*R*_free_ † (%)	19.05
R.m.s.d., bond lengths (Å)	0.011
R.m.s.d., bond angles (°)	1.20
Average *B* factors (Å^2^)	
Protein atoms (molecule *A*/*B*)	36.7/44.4
Heavy metals (molecule *A*/*B*)	31.2/37.1
Lipid and detergents	68.2
Waters	58.4
All atoms	44.2
No. of amino-acid residues	
Total	3614
Determined	3550
Multiple conformation	125

† 5% of the total reflections were used for the calculation.

**Table 3:** Occupancies and *B* factors of O atoms in the Mg-containing water clusters for the pH 7.3 and pH 5.7 structures of oxidized-form CcO

	pH 7.3 oxidized form			pH 5.7 oxidized form†		
Name	Residue No./chain ID	*B* factor (Å^2^)	Occupancy	Residue No./chain ID	*B* factor (Å^2^)	Occupancy
1	1/*b*	26.3	1.00	733/*A*	22.0	1.00
2	2/*b*	25.2	1.00	817/*A*	20.3	1.00
3	3/*b*	27.3	1.00	863/*A*	22.2	1.00
4	4/*b*	26.6	1.00	793/*A*	23.3	1.00
5	5/*b*	25.5	1.00	823/*A*	20.0	1.00
6	6/*b*	26.8	1.00	859/*A*	21.3	1.00
7	7/*b*	29.7	1.00	447/*B*	23.4	1.00
8	8/*b*	25.8	1.00	521/*B*	21.8	1.00
9	9/*b*	27.5	1.00	486/*B*	22.8	1.00
10	10/*b*	28.1	1.00	721/*A*	21.6	1.00
11	11/*b*	28.1	1.00	902/*A*	22.8	1.00
12	12/*b*	26.0	1.00	811/*A*	21.9	1.00
13	13/*b*	26.6	1.00	848/*A*	21.2	1.00
14	14/*b*	27.5	1.00	835/*A*	22.7	1.00
15	15/*b*	28.6	1.00	728/*A*	22.4	1.00
16	16/*b*	26.8	0.90	741/*A*	21.8	0.75
17	17/*b*	27.5	0.90	473/*B*	22.1	0.80
18	18/*b*	27.7	0.75	901/*A*	22.0	0.65
19_A	19a/*b*	27.3	0.70	492a/*B*	21.9	0.60
19_B	19b/*b*	26.6	0.25	492b/*B*	23.2	0.40
20	20/*b*	28.2	0.95	791/*A*	21.8	0.90
21	21/*b*	26.9	0.80	499/*B*	22.1	0.75
22	22/*b*	28.9	0.20	441/*B*	21.9	0.30
23	23/*b*	28.2	0.70	570/*B*	22.4	0.60
Total			21.15			20.75

† PDB entry is 5b1a.
